# Seasonal Occurrence of Cattle Fascioliasis in Kelantan, Malaysia

**DOI:** 10.3390/vetsci10030202

**Published:** 2023-03-07

**Authors:** Arizam Muhammad Faez, Mohamad Ahmad Najib, Abdul Ghafar Noraini, Wong Weng Kin, Aziz Abd Rahman, Wan Abdul Wahab Wan Nor Amilah, Noor Jamil Noor Izani

**Affiliations:** 1 Faculty of Veterinary Medicine, Universiti Malaysia Kelantan, Pengkalan Chepa, Kota Bharu 16100, Kelantan, Malaysia; 2 School of Health Sciences, Universiti Sains Malaysia, Health Campus, Kubang Kerian, Kota Bharu 16150, Kelantan, Malaysia

**Keywords:** cattle, climatic factors, fascioliasis, prevalence, seasonal, Kelantan

## Abstract

**Simple Summary:**

Fascioliasis is a common disease of cattle, but it remains a neglected disease in Malaysia. Several studies have been published in the past to address the significant economic losses to the livestock industry due to fascioliasis, but very few have investigated the effect of climatic factors in the occurrence of the disease. We performed a longitudinal study by examining 40 cattle on a monthly basis for a period of 12 months. In addition, the effect of climatic factors such as temperature, humidity, rainfall, and pan evaporation in the disease occurrence were analysed by Pearson’s correlation. We found that cattle fascioliasis is prevalent in Kelantan, and the occurrence of cattle fascioliasis was positively correlated with rainfall and humidity and negatively correlated with evaporation. These observations suggest that the implementation of preventive strategies during monsoon season, which has higher rainfall and humidity and lower evaporation, should be considered to effectively control cattle fascioliasis in Kelantan.

**Abstract:**

A longitudinal study was conducted in five randomly selected farms in Kelantan, Malaysia to determine the seasonal occurrence of cattle fascioliasis and its association with climatic factors. A total of 480 faecal samples were collected by a random purposive sampling method from July 2018 to June 2019. The faecal samples were examined for the presence of *Fasciola* eggs using a formalin ether sedimentation technique. Meteorological data including temperature, humidity, rainfall, and pan evaporation were obtained from a local meteorological station. The overall prevalence of cattle fascioliasis in Kelantan was 45.8%. The prevalence was observed to be slightly higher during the wet season from August to December (50–58%) than during the dry season from January to June (30–45%). Meanwhile, the mean eggs per gram (EPG) were highest in June (191.1 ± 0.48) and lowest in October (77.62 ± 95.5). However, there were no significant differences in the mean of EPG between the monthly prevalence, tested using one-way ANOVA (*p* = 0.1828). A statistically significant association (*p* = 0.014) was observed between the disease and cattle breeds, with Charolais and Brahman showing lower odds of having the disease. There were significant moderate-to-strong positive correlations between cattle fascioliasis and rainfall (r = 0.666; *p* = 0.018) and humidity (r = 0.808; *p* = 0.001), as well as strong negative correlations with evaporation (r = −0.829; *p* = 0.001). The results indicated that the higher prevalence of cattle fascioliasis in Kelantan was correlated with the climatic factors, which include higher rainfall and humidity and lower evaporation.

## 1. Introduction

Fascioliasis is a neglected zoonotic disease caused by *Fasciola hepatica* and *Fasciola gigantica* predominantly found in ruminants and wildlife. *F. hepatica* is normally found in the temperate region, and *F. gigantica* is more common in tropical countries [1]. Recent studies have demonstrated that fascioliasis generates considerable financial losses in the cattle business [2,3]. The infestation causes huge economic losses through cattle’s bodyweight loss, condemnation of liver, decreased milk production, increase in the cost of anthelmintic treatment, and a reduction in animals’ fertility and mortality [4,5,6]. Globally, the production losses due to fascioliasis in livestock were estimated to be over USD 3 billion annually [7]. In Malaysia, an alarming occurrence of cattle and other ruminant fascioliasis has been reported in several states in the peninsular Malaysia as well as in Sabah [8,9].

Cattle become infected mainly by consuming metacercarial cysts found in the soil, grass, and aquatic plants, as well as in forage and contaminated drinking water [10]. Climatic and environmental factors may cause important variations in the level of exposure to *Fasciola* spp. and the development of the parasite’s intermediate molluscan and free-living stages [11]. Climate change may increase rainfall and humidity in some areas, creating new habitats for *Fasciola* and its intermediate hosts. The intermediate hosts, such as *Galba truncatula* and *Radix natalensis,* are responsible for the majority of *Fasciola* transmission worldwide [12]. By producing a large number of cercariae from a single miracidium, snail infection increases production of the *Fasciola* infective stage. The free-swimming cercariae then disperse away from the intermediate host and encyst into metacercariae, which remain in watery areas or attach to aquatic plants until eaten by the mammal’s host.

Fascioliasis has been reported in Kelantan, a northeastern state of peninsular Malaysia having a climate similar to southern Thailand with wet and long dry seasons [13]. The cross-sectional study showed 14.6% of the cattle examined were positive for fascioliasis based on faecal examination. The need for implementation of effective control for fascioliasis in Kelantan is crucial. In this regard, seasonal surveillance plays a significant role to identify the trend of fascioliasis incidence and the association of the disease with climatic and environmental factors. Furthermore, assessing the situation of fascioliasis raises awareness of the parasites’ importance to livestock productivity as well as their zoonotic potential [14]. In the absence of regional information on the prevalence of cattle fascioliasis in Kelantan, as well as the seasonal trends for the infestation, this surveillance becomes necessary. Therefore, the purpose of this study was to determine the seasonal prevalence of cattle fascioliasis in Kelantan, as well as the effect of climatic factors associated with the disease. Such information will be beneficial to improve understanding of the occurrence of cattle fascioliasis in Kelantan so that effective strategies can be designed for its control.

## 2. Materials and Methods

### 2.1. Ethical Approval

The present study protocol was reviewed and approved by the Animal Ethics Committee of Universiti Sains Malaysia (AECUSM). A certificate of approval was issued with the registration number USM/IACUC/2017(107) (852).

### 2.2. Study Design

**Figure 1:**
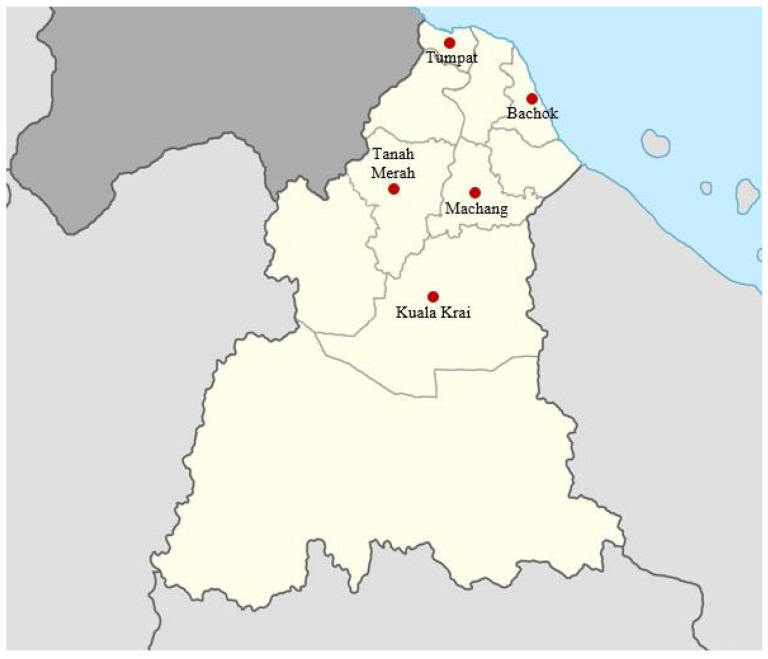
Map of Kelantan, Malaysia with five red circles showing locations of five sampling areas.

This longitudinal study was carried out in five regions of Kelantan, Malaysia: the north (Tumpat, Kelantan, Malaysia), south (Kuala Krai, Kelantan, Malaysia), east (Tanah Merah, Kelantan, Malaysia), west (Bachok, Kelantan, Malaysia), and central region of Kelantan (Machang, Kelantan, Malaysia). One farm was selected from each region. The farms were chosen using a random purposive sampling method, with only farms that met the selection criteria being visited. The selection criteria included having a proper animal restrainer and having at least 10 cattle over the age of 6 months on the farm. The selected study areas were designated at Kampung Simpangan, Tumpat (6.21313, 102.08039), Kampung Paloh, Tanah Merah (5.87437, 102.16576), Kampung Melawi, Bachok (5.99697, 102.41254), Batu Mengkebang, Kuala Krai (5.61426, 102.19158), and Kg. Ulu Sat, Machang (5.7769, 102.28642) (Figure 1).

### 2.3. Samples Collection

A total of 480 samples were collected from July 2018 until June 2019. Prior to coprological examination, fresh faecal samples were collected from the rectum of the cattle and transferred into labelled 60 mL stool containers. Cattle profiles, including age, breed, and sex, were created. The cattle’s ages were determined using farm records. The cattle’s gender was determined by examining the presence of sexual organs. The cattle breed was decided after consultation with the farm owners.

### 2.4. Meteorological Data

Meteorological data, including maximum and minimum temperature (°C), relative humidity (%), rainfall (mm), and pan evaporation (mm) was obtained from the meteorological station of Kelantan, Malaysia. The meteorological data obtained in each month was calculated by averaging the daily data recorded.

### 2.5. Coprological Examination

**Figure 2:**
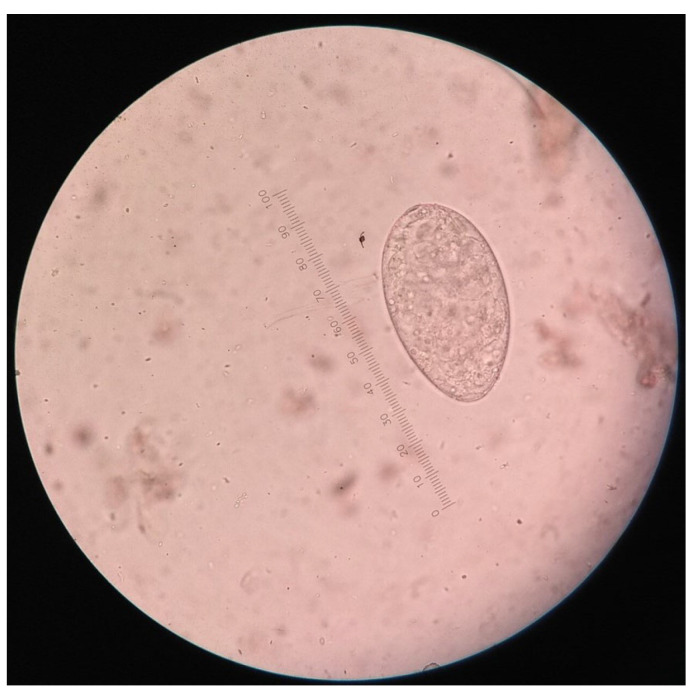
Photomicrograph of *Fasciola* egg viewed using light microscope at 400× magnification.

For the detection of *Fasciola* spp. eggs, faecal samples were subjected to the formalin-ether sedimentation technique [4]. A light microscope with an eyepiece reticle was used to examine the sediment at 100× and 400× magnification (Zeiss, Germany). The egg of *Fasciola* spp. was identified using the morphology described in a previous study [15,16]. Eggs of *Fasciola* spp. were golden in colour, ellipsoidal, operculated, and measured 130–150 µm long by 60–90 µm wide (Figure 2). The presence of *Fasciola* spp. eggs in the faecal samples was used to interpret a positive result for *Fasciola* spp. infestation. The McMaster egg-counting technique was applied to positive samples to determine the number of EPG. The load of infestation was categorized into three levels, i.e., mild infestation (1–500 EPG), moderate infestation (501–1000 EPG), and severe infestation (above 1000 EPG) [17].

### 2.6. Statistical Analysis

SPSS version 24.0 (IBM Corporation, New York, NY, USA) was used to perform statistical analysis on the data. The overall prevalence, monthly prevalence, and geographical distribution of the infestation was calculated and expressed as a percentage (%). The difference in the mean of the EPG between positive samples and the monthly prevalence was compared using one-way ANOVA. Pearson’s Chi-square (χ^2^) was used to determine the relationship between *Fasciola* spp. infestation and cattle profiles. At 95% confidence intervals (CI) of the odds ratio, the degree of association between the prevalence and the associated factors was determined using binary logistic regression analysis. The presence of a linear relationship between the prevalence of fascioliasis and meteorological factors was analysed by Pearson’s correlation. A statistically significant between-variables value was considered if the calculated *P*-value was less than 0.05 at a 95% confidence interval (CI).

## 3. Results

### 3.1. Characteristics of Cattle

Of the 480 cattle, 306 (63.7%) were female, and 174 (36.3%) were male. The cattle’s age ranged from 1 year to 5 years, with a median of 2 years. A total of 224 (46.7%) cattle were of the Kedah-Kelantan breed (local breed), 188 (39.2%) were Charolais, and the remaining were Brahman (33 (6.9%)), Limousine (32 (6.7%)), and Blonde (3 (0.6%)) (Table 1).

### 3.2. Monthly Trend of the Infestation and EPG

**Figure 3:**
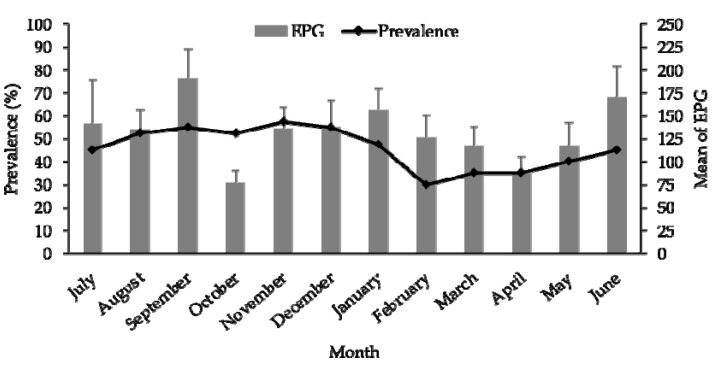
Monthly trend of cattle fascioliasis in Kelantan from July 2018 to June 2019.

The overall prevalence of fascioliasis was 45.8% (220/480), of which 98.6% (217/220) had mild infestation, and 1.4% (3/220) had moderate infestation. The monthly prevalence ranged from 30% to 57.5%, with the peak observed in November 2018 (Figure 3). The monthly trend of cattle fascioliasis showed an increasing trend from June 2018 (45%) to November 2018 (57.5%). The prevalence gradually decreased beginning in December 2018 (55%), and was the lowest in February 2019 (30%). It again increased from March 2019 to June 2019. The mean EPG identified from cattle infected with *Fasciola* spp. ranged from 77.62 ± 13.1 (October 2018) to 191.1 ± 31.49 (September 2018). There were no significant differences between the monthly prevalence and mean of the EPG as tested using ANOVA (*p* = 0.1828).

### 3.3. Geographical Distribution of Cattle Fascioliasis

The prevalence of cattle fascioliasis was observed to be the highest in Tumpat (62.5%), followed by Kuala Krai (53.1%) and Machang (47.9%). Meanwhile, Tanah Merah and Bachok recorded the lowest prevalence of 33%.

### 3.4. Association between Cattle Profiles and Fascioliasis

Pearson’s Chi-square analysis showed that there were no significant association between the infestation and age and sex (Table 1). Concerning cattle breeds, there was a significant association between the infestation and cattle breeds (*p* = 0.014). Charolais (OR: 0.523; 95% CI: 0.353, 0.777) and Brahman (OR: 0.529; 95% CI: 0.247, 1.135) cattle showed a lower odd of having *Fasciola* spp. infestation as compared to Kedah-Kelantan cattle.

### 3.5. Correlation of Cattle Fascioliasis with Climatic Factors

**Figure 4:**
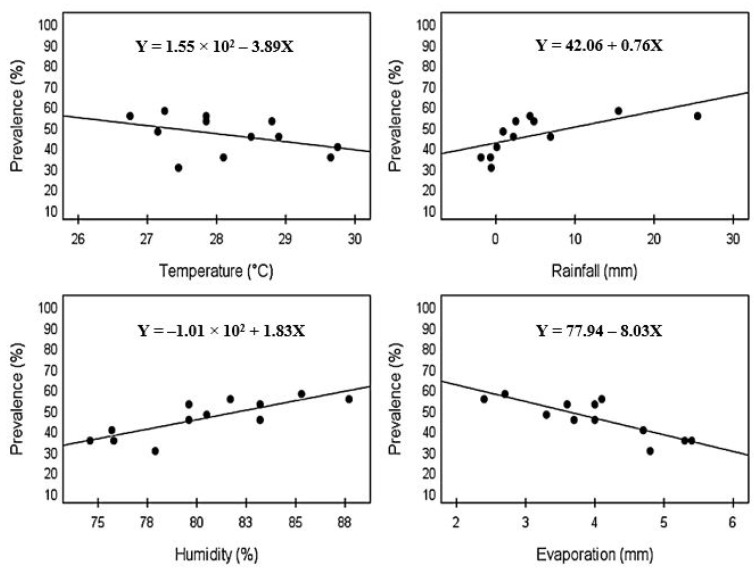
Scatter plot depicting the correlations between cattle fascioliasis and temperature (°C), rainfall (mm), humidity (%), and evaporation (mm).

The Pearson’s correlation analysis between cattle fascioliasis and climatic factors is depicted in Figure 4. The analysis showed no significant correlation of cattle fascioliasis with temperature (r = −0.414; *p* = 0.181). On the contrary, there were significant correlations between cattle fascioliasis and rainfall, humidity, and evaporation. The prevalence of cattle fascioliasis in Kelantan showed significant moderately strong positive correlations with rainfall (r = 0.666; *p* = 0.018) and strong positive correlations with humidity (r = 0.808; *p* = 0.001). The prevalence of cattle fascioliasis increased as the rainfall and humidity increased. Meanwhile, a strong significant negative correlation was observed between cattle fascioliasis and evaporation (r = −0.829; *p* = 0.001). As the evaporation increased, the prevalence of cattle fascioliasis decreased.

## 4. Discussion

Investigating the seasonal occurrence of cattle fascioliasis is important to improve understanding of the seasonal pattern of the disease. The present study is the first to determine seasonal occurrence of cattle fascioliasis in Kelantan and its correlation with climatic factors. The overall estimated prevalence of cattle fascioliasis in this study was 45.8%, higher than the previous cross-sectional surveillance of cattle fascioliasis in Kelantan, which reported an overall prevalence of 14.6% based on coprological examination of the *Fasciola* eggs [13]. The high prevalence of cattle fascioliasis may be due to irregular anthelmintic treatment in the investigated areas. In addition, most of the farms were in rural areas located near the river, which is the main source of the cattle’s drinking water [13]. The monthly average EPG trends showed the prevalence peaked in November 2018, which coincided with the wet season that could support the survival of metacercarial cyst for ingestion during grazing.

Even though it was previously reported that when prevalence increases, egg count also increases, indicating the increase in worm burden on infected hosts [18], the present study showed no significant differences between the monthly prevalence and the mean EPG. According to an observational study in Cuba, the severity of the infestation was significantly associated with the body condition of the animals rather than climatic factors [19]. Those cattle with good body condition showed a significantly lower mean of EPG. Of the five sampling areas, cattle in Tumpat and Kuala Krai were the most affected, with an overall prevalence of 62.5% and 53.1%, respectively. These two areas are located near to rivers, which saw overflowing and flooding during the wet season and provide an abundance of vegetation, which favours the environmental condition for the *Fasciola* life cycle. In addition, the vegetation is used as animal feed during mostly the wet season when the animals are kept from roaming. This factor may explain the high prevalence of cattle fascioliasis in these two areas.

The study of the association between cattle profiles and fascioliasis showed no significant association between the infestation and age. However, the current findings contradicted previous South African studies that found a lower prevalence in older cattle [17]. The low prevalence in older cattle was attributed to the parasite’s high immunogenicity, which facilitates the stimulation of acquired immunity in older animals. Furthermore, the current study found no significant relationship between the infestation and sex. This finding, however, contradicted the findings of an Ethiopian study, which found a significantly higher incidence in females [20]. This could be because most female cattle were kept for milking the young, which is a stressful physiological factor that may affect their immunity against the infestation [21]. On the other hand, there was a significant association between the infestation and cattle breed. Charolais and Brahman cattle showed lower odds of having *Fasciola* spp. infestation as compared to the Kedah-Kelantan cattle. One plausible reason could be that Charolais and Brahman were kept in healthier and more nourishing conditions than other breeds, as these two are more expensive.

The study of climatic factors showed that there were significant positive correlations between cattle fascioliasis and rainfall and humidity and negative correlations with evaporation. These results were in agreement with a systematic review on prevalence and epidemiological risk factors of fascioliasis in Bangladesh, which reported a higher prevalence in the rainy season [22]. During the rainy season, the watery area and humidity favours the growth of snails, which are intermediate hosts for the miracidia to develop into cercariae. Owing to this, the availability of metacercaria were abundant during that season, exposing the cattle to a higher risk of obtaining the infestation [23].

Regarding the effect of temperature, a similar finding was observed in Pakistan, in which no significant correlation was recorded between cattle fascioliasis and this factor [18]. Nevertheless, these results were in disagreement with those reported in Ireland and Mexico, where it was found that temperature was a positive predictor of cattle fascioliasis [11,24]. One plausible reason attributed to the contradictory finding with these studies may be due to huge variations in temperature in those areas. During summer, temperatures are high, and the soil moisture becomes deficient, thus threatening the intermediate hydrophilic stages of the parasite’s life cycle [25].

Regular surveillance of the cattle fascioliasis occurrence should be carried out in Kelantan. Such surveillance will improve our understanding of the effects of climatic factors on the disease exposure and provide information that can help us to develop effective control and prevention strategies. Thus far, there is no molecular prevalence study on fascioliasis in the study areas. Such studies would shed light on the presence of either mixed or mono-species *Fasciola* infestation and enable the identification of the predominant *Fasciola* species in the study areas. Furthermore, future investigation of the intermediate host by morphological and molecular techniques is needed to enable the identification of the host species responsible for *Fasciola* spp. transmission in these areas.

## 5. Conclusions

The present longitudinal study unravelled the seasonal occurrence of cattle fascioliasis in Kelantan. A slightly higher prevalence of cattle fascioliasis was observed in the monsoon season as compared to the dry season. The etiological study of risk factors showed a significant association between cattle fascioliasis and cattle breed, in which Charolais and Brahman showed lower odds of having the infestation. A higher prevalence of cattle fascioliasis in Kelantan was significantly correlated with climatic factors such as rainfall, humidity, and evaporation. Owing to the economic importance of fascioliasis, there is a need for the implementation of effective strategies to control cattle fascioliasis in Kelantan.

## Figures and Tables

**Table 1:** Risk factors associated with cattle fascioliasis in Kelantan, Malaysia.

Attribute	Overall *n* (%)	Positive *n* (%)	Negative *n* (%)	OR (95% CI)	*p* Value
Samples	480 (100%)	220 (45.8%)	260 (54.2%)		
Age (Year)					0.358
1	98 (20.4%)	39 (39.8%)	59 (60.2%)		
2	152 (31.7%)	78 (51.3%)	74 (48.7%)		
3	134 (27.9%)	58 (43.3%)	76 (56.7%)		
4	82 (17.1%)	37 (45.1%)	45 (54.9%)		
5	14 (2.9%)	8 (57.1%)	6 (42.9%)		
Sex					0.063
Male	174 (36.3%)	70 (40.2%)	104 (59.8%)		
Female	306 (63.7%)	150 (49.0%)	156 (51.0%)		
Breed					0.014 *
Kedah-Kelantan	224 (46.7%)	119 (53.1%)	105 (46.9%)		
Charolaise	188 (39.2%)	70 (37.2%)	118 (62.8%)	0.523 (0.353, 0.777)	
Brahman	33 (6.9%)	12 (37.5%)	20 (62.5%)	0.529 (0.247, 1.135)	
Limousine	32 (6.7%)	18 (54.5%)	15 (45.5%)		
Blonde	3 (0.6%)	1 (33.3%)	2 (66.7%)		

Note: Pearson χ^2^ analysis, * statistically significant at *p* value < 0.05. OR: odds ratio, CI: confidence interval.

## Data Availability

Not applicable.
